# Supplementary figures and images for: A transcription-centric model of SNP-age interaction
Source: PLoS Genet. 2021 Mar 26;17(3):e1009427. doi: 10.1371/journal.pgen.1009427 (PMC7997000; doi:10.1371/journal.pgen.1009427)

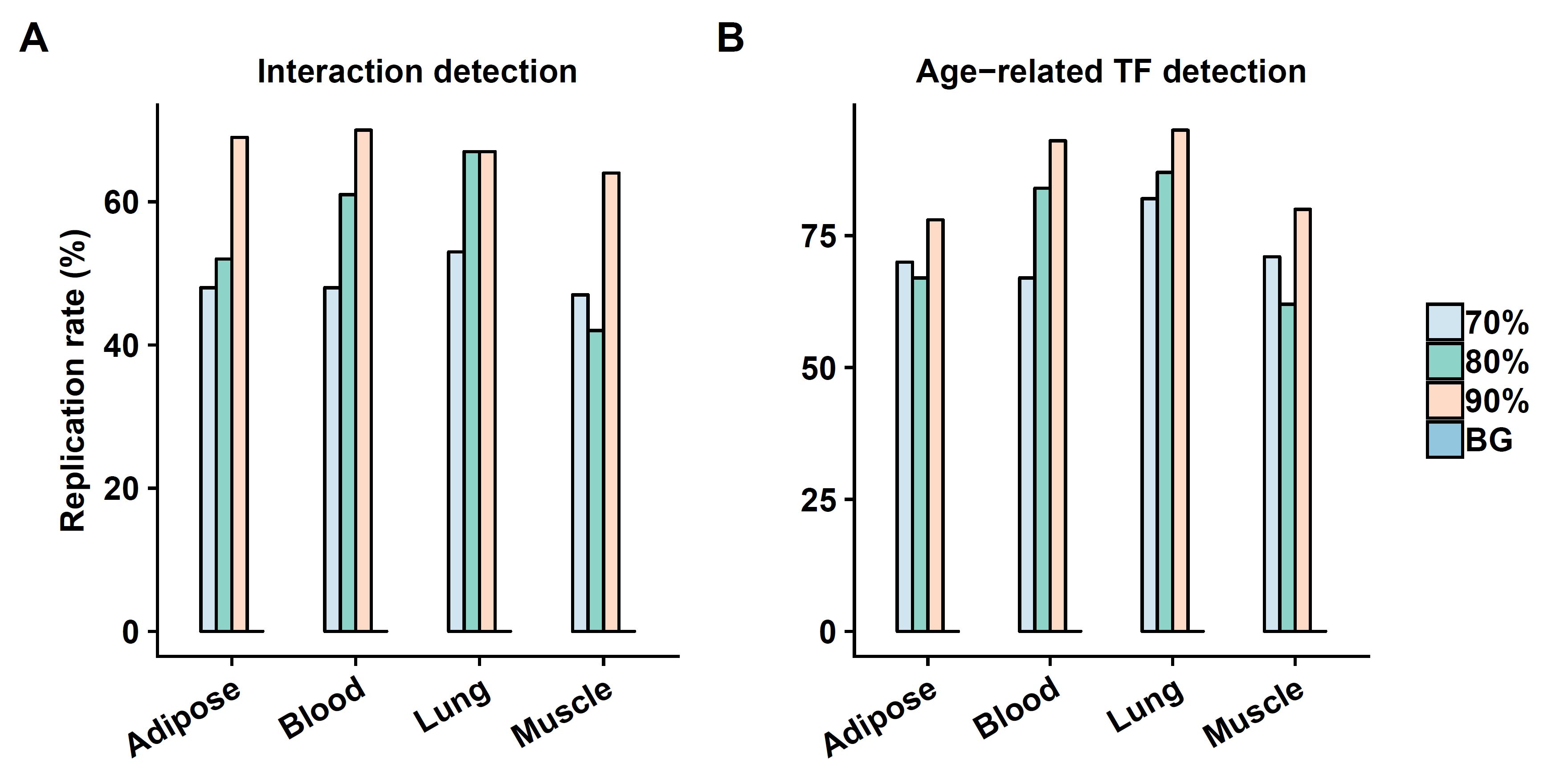

Supplement: S1 Fig — (A) bar plot depicting the replication rates of interactions across 4 tissues. (B) bar plot depicting the replication rates of age-associated TFs across 4 tissues. The y-axis represents the replicate rate (%) relative to the original data, while x-axis denotes the tissues. The bars in four colors respectively represent the performance for the 70%, 80%, 90% down sampling and randomly shuffled background data. (TIF) [file pgen.1009427.s001.tif]

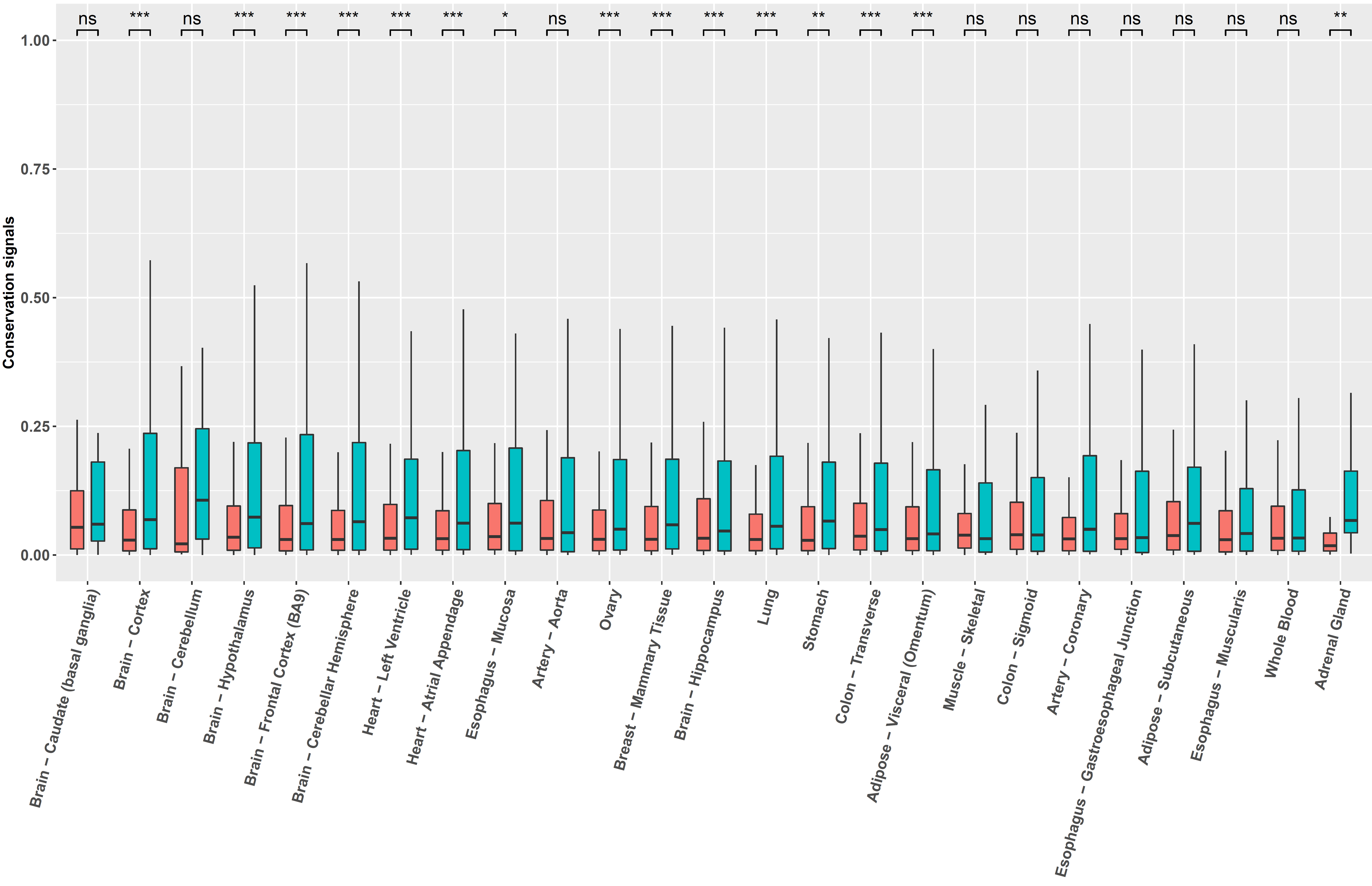

Supplement: S2 Fig — The y-axis denotes the conservation score, while the x-axis represents tissues. The green denotes the detection interaction relevant SNPs and the red denotes random SNPs without passing DNase filtration. (TIF) [file pgen.1009427.s002.tif]

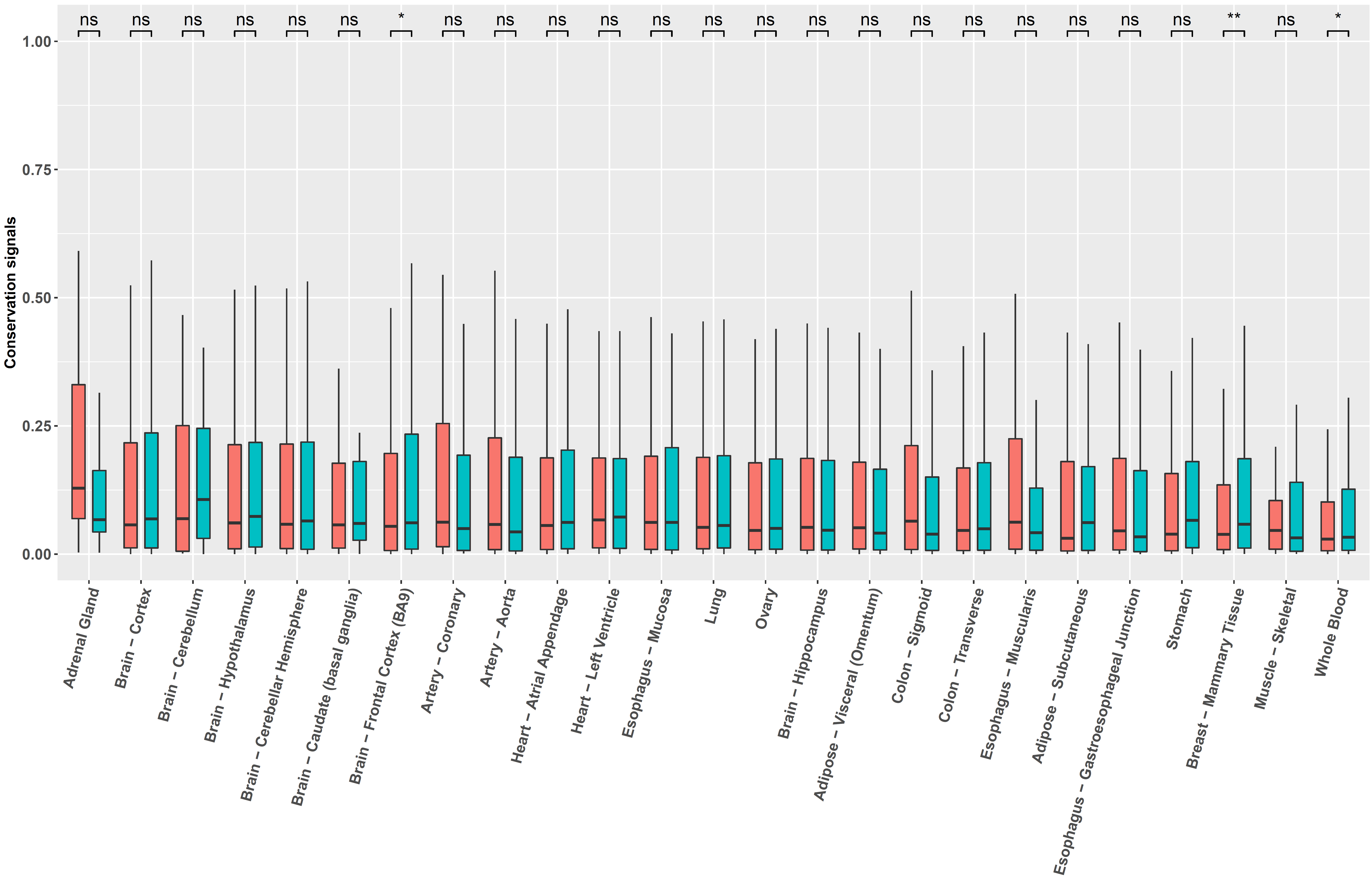

Supplement: S3 Fig — The y-axis denotes the conservation score, while the x-axis represents tissues. The green bar denotes the detection interaction relevant SNPs and the red bar denotes random SNPs which passed DNase filtration. (TIF) [file pgen.1009427.s003.tif]

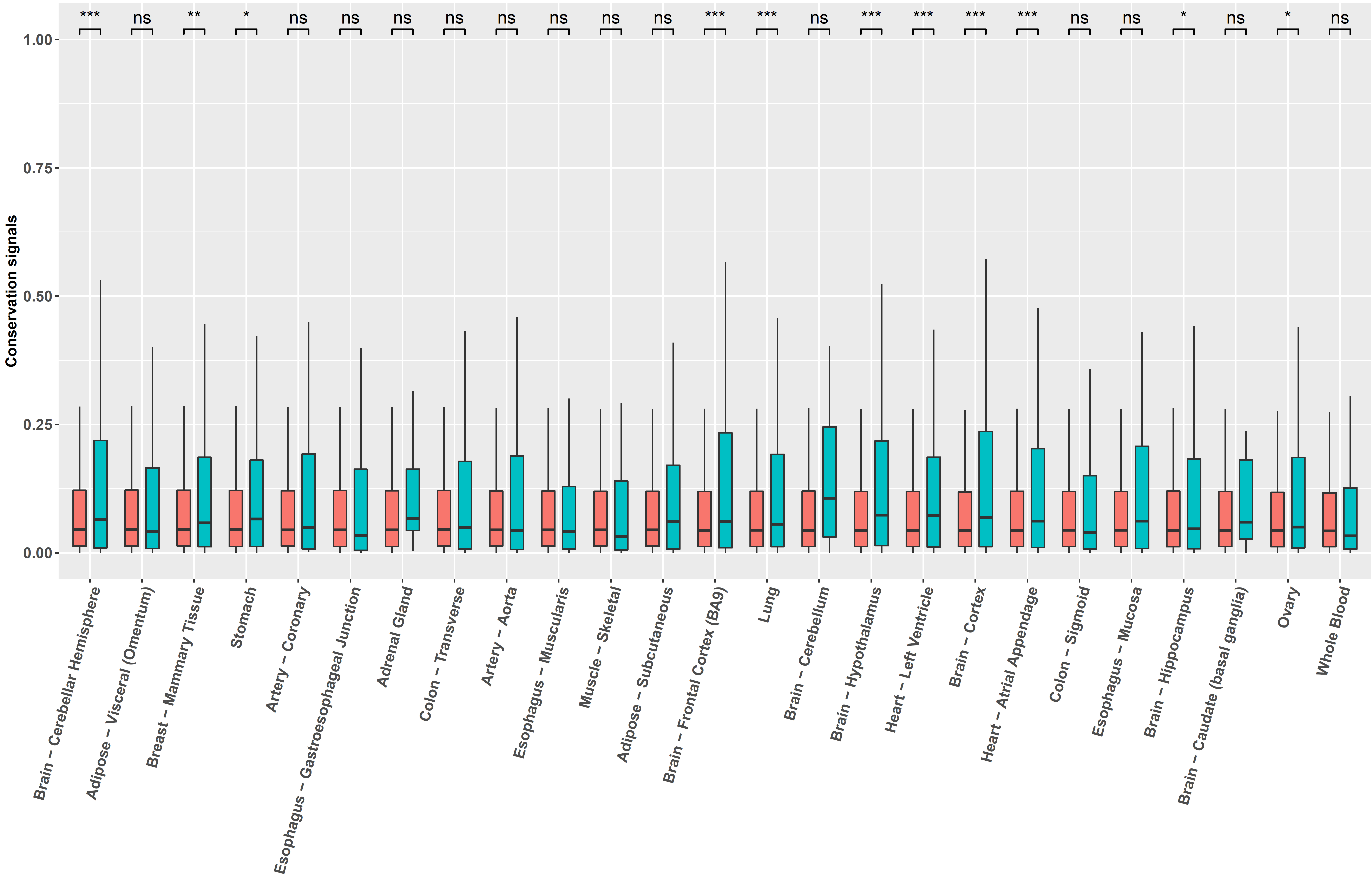

Supplement: S4 Fig — The y-axis denotes the conservation score, while the x-axis represents tissues. The green bar denotes the detection interaction relevant SNPs and the red bar denotes eSNPs. (TIF) [file pgen.1009427.s004.tif]

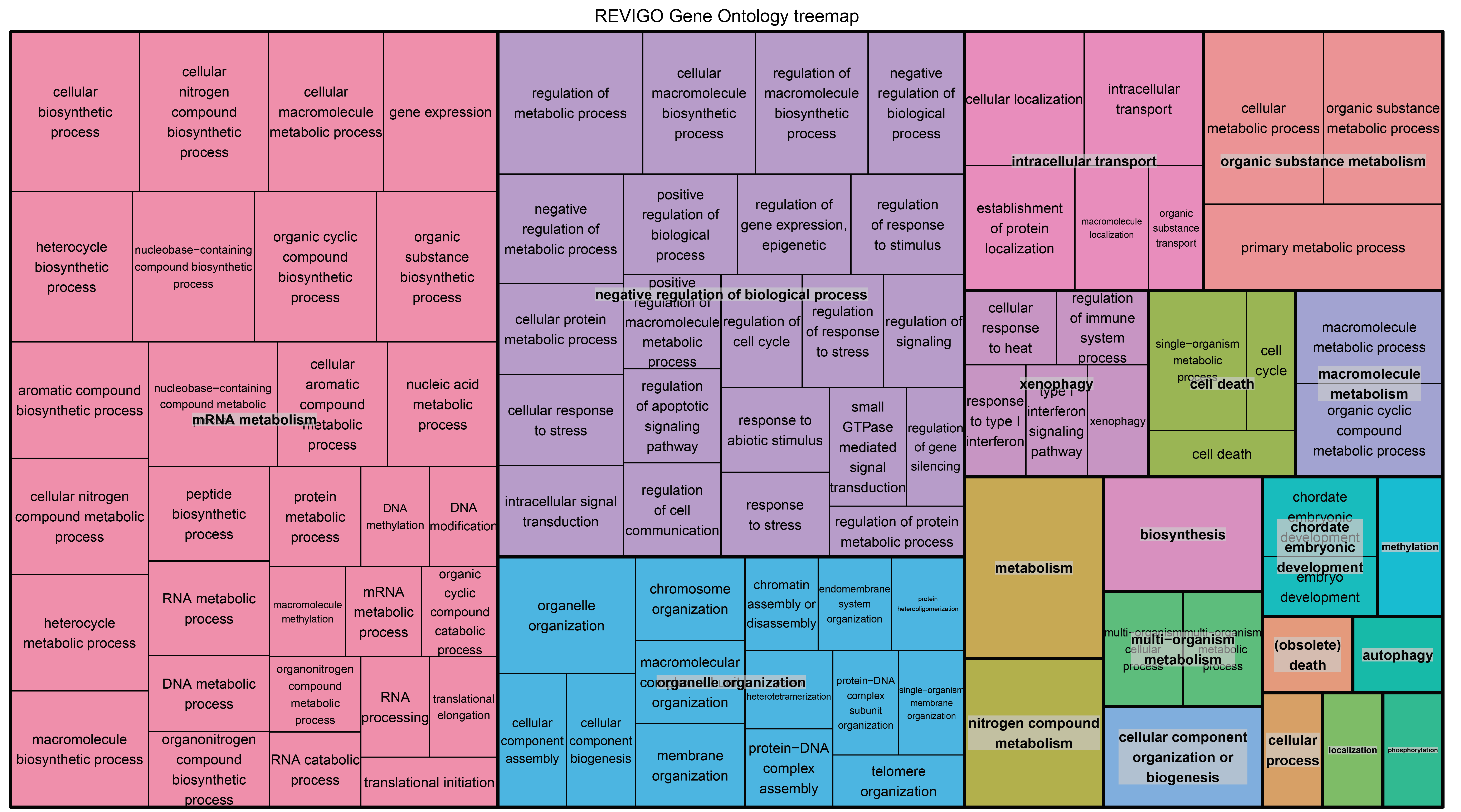

Supplement: S5 Fig — Enrichment analysis was done independently in each tissue, and the tree-map view shows the GO terms that were enriched (FDR ≤ 5%) in at least three tissues. (TIF) [file pgen.1009427.s005.tif]

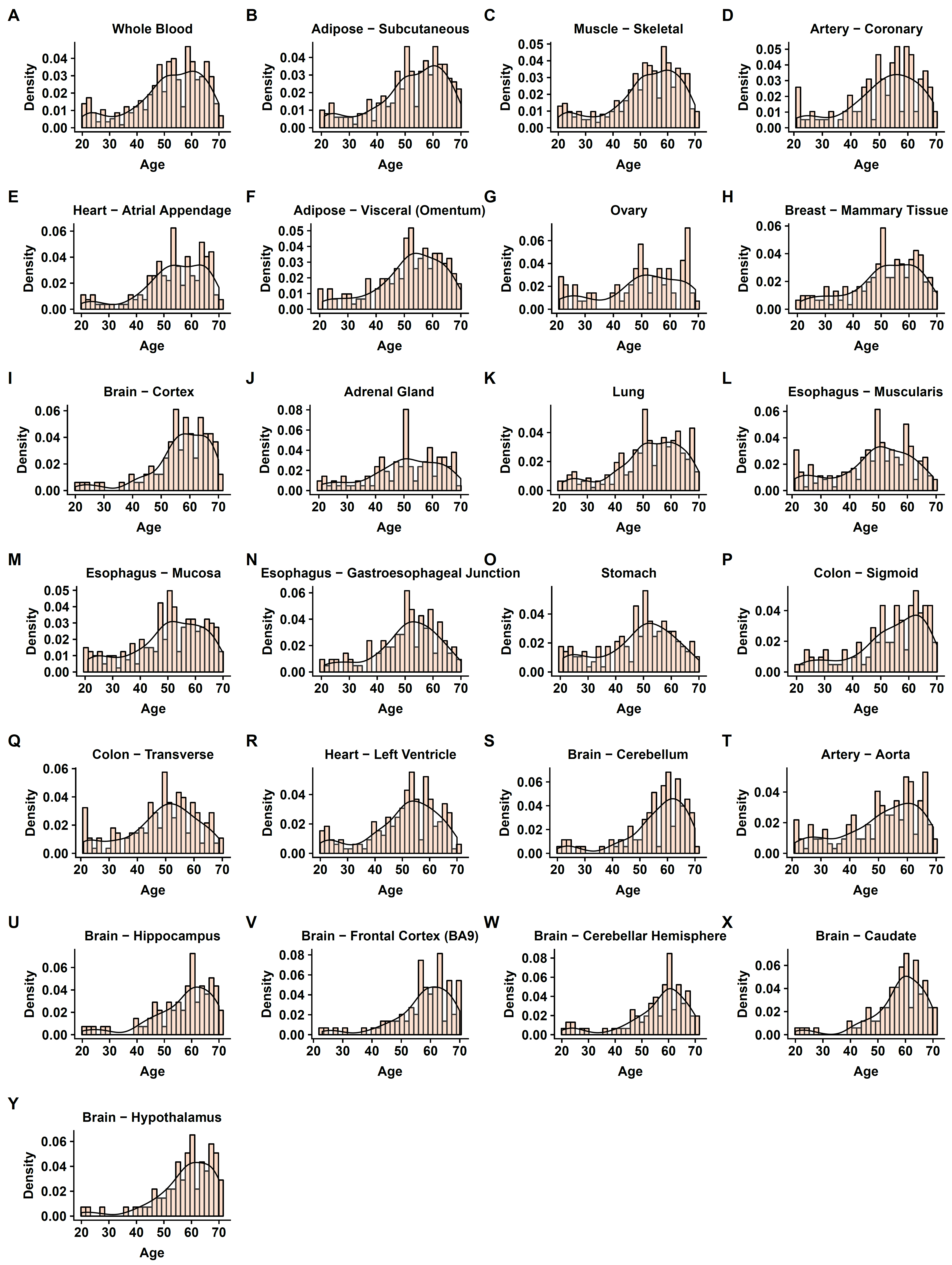

Supplement: S6 Fig — (A-Y) histograms depicting the age distribution in each of the 25 tissues. (TIF) [file pgen.1009427.s006.tif]

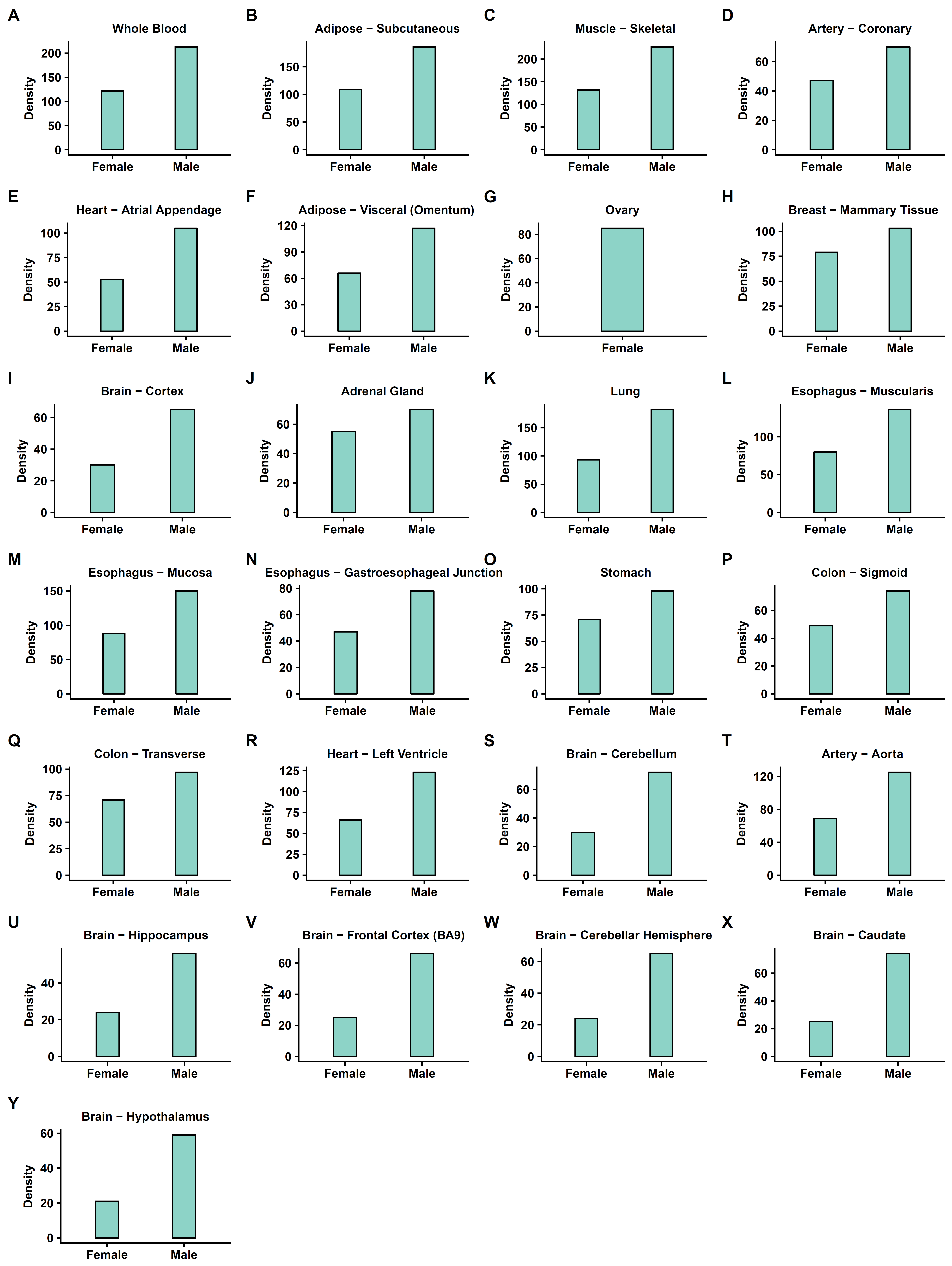

Supplement: S7 Fig — (A-Y) histograms depicting the sex distribution in each of the 25 tissues. (TIF) [file pgen.1009427.s007.tif]

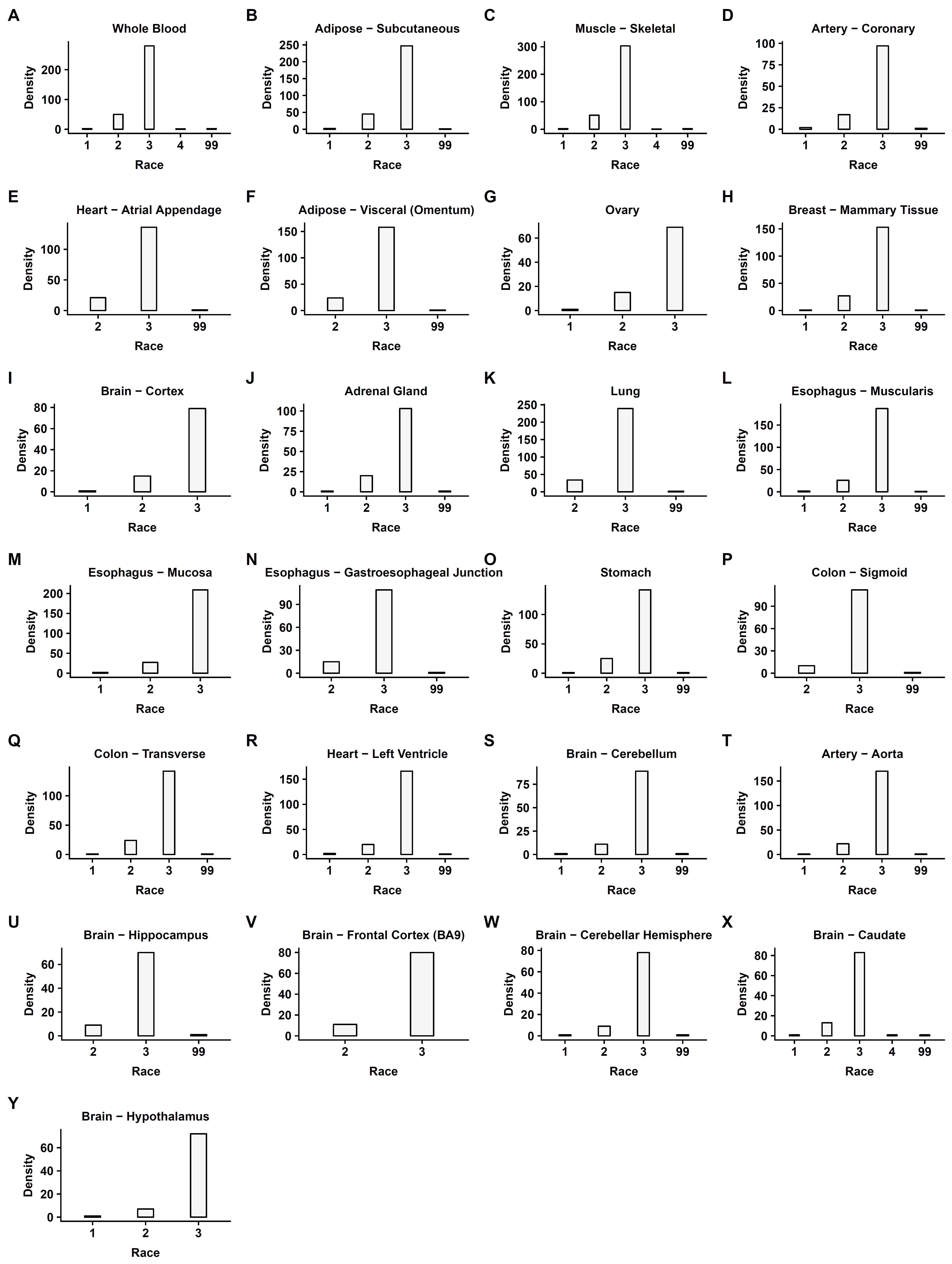

Supplement: S8 Fig — (A-Y) histograms depicting the race distribution in each of the 25 tissues. For race, “1”: Asian, “2”: African American, “3”: white, “4”: American Indian, “98”: Not Reported, “99”: Unknown. (TIF) [file pgen.1009427.s008.tif]
